# Study of Drug-Drug Interactions in Prescriptions of General Practitioners and Specialists in Iran 2007-2009

**Published:** 2011

**Authors:** Fariba Ahmadizar, Fatemeh Soleymani, Mohammad Abdollahi

**Affiliations:** a *RUD Committee, Deputy for Food and Drug Affairs, Ministry of Health and Medical Education, Tehran, Iran.*; b *Faculty of Pharmacy and Pharmaceutical Sciences Center, Tehran University of Medical Sciences, Tehran, Iran.*

**Keywords:** Drug-drug interactions, Clinical significant, Polypharmacy, Patient

## Abstract

Prescriptions written by general practitioners and medical specialists were studied and compared to determine the type, time of onset and clinical importance of drug-drug interactions (DDIs) in an attempt to reduce further complications.

In 2007, 28, 956, 638 prescriptions and 15, 610, 912 prescriptions in 2008 were filled by pharmacies affiliated with medical science universities. These prescriptions, prescribed by physicians from 33 Iranian medical universities nationwide were then evaluated with a prescription processing software named Pardazesh Nosakh. After processing and analyzing the data, DDIs were discovered in 14 different medical specialists consisting of internists, cardiologists, neurologists, psychiatrists, neurosurgeons, general surgeons, infectious diseases, urologists, dermatologists, ENT, ophthalmologists, orthopedists, and pediatrician. The results were then analyzed through methods applied in the book of Drug Interaction Facts.

The results revealed that in 2007-2008, 0.77% of prescriptions had DDIs out of which 0.67% were with significant clinical importance. The percentage of interactions with significant clinical importance was higher in prescriptions of medical specialists and of those, cardiologists and internists ranked top on the list, while dermatologists ranked the lowest. The most common interacting combination prescribed was digoxin and furosmide in 2007-2008, and captopril and triamteren in 2008-2009. Moreover, this study showed that polypharmacy was an important factor which led to DDIs. Drug interactions were common among outpatients prescribed multiple medications and the rate of DDIs increased with the number of drugs prescribed.

It is our opinion that by being up-to-date on drug information and participating in related educational classes and workshops, physicians can increase the chances of choosing the correct drug treatment and hence significantly decrease possible DDIs side effects.

## Introduction

Among medical errors, potentially serious drug-drug interactions (DDIs) have recently received increased attention. Currently available estimates of DDI incidences vary widely depending on the method of defining and finding potential DDIs and the method of defining the population assessed. Published studies have reported proportions of potential DDIs ranging from 2.2% to 30% in hospitalized patients and from 9.2% to 70.3% in ambulatory patients (1-3).

A DDI can be defined as a pharmacological or clinical response to the administration of a drug combination, different from that of anticipated one from the known effects of the two agents when given alone. The clinical result of a DDI may be manifested as antagonism, synergism, or idiosyncratic (4).

The consequences of mistakes and drug errors such as drug interactions affect millions of patients every year and contribute to 5% of patient admissions into hospitals (5-8). These medical errors also increase the patients› expenses, which ultimately affects the whole society (9).

There is little knowledge in terms of the epidemiology of DDIs on the clinical level and most evidence and documentations on this come from case reports, voluntary studies and/or through reports from DDIs detected in admitted hospital patients (10-16).

The treatment of a disease usually requires the use of more than one drug. When patients have multiple symptoms, it becomes necessary to prescribe a number of drugs. In this case, physicians must consider the possibility of DDIs. DDIs mostly occur among drugs with a low therapeutic index having a small difference between their therapeutic and toxic or lethal dose. This means, with the slightest change in the dosage of a drug, it can produce dangerous and harmful effects. The severity of illness in the patient being treated is also another predisposing factor to DDIs, such that treating cardiovascular, collagen vascular, and infectious disease and psychiatric disorders have the greatest potential for dangerous drug interactions.

Drug interactions are one of the most important drug mistakes known and are only predictable and preventable by revision of previous documentations, reports, and clinical studies (8). However, most physicians are unaware of major and clinically important drug interactions (17-21); thus, equipping physicians› clinics with a computerized physician order entry (CPOE) system can warn physicians of impending drug interactions, and should this system be further supervised by pharmacologists, especially focusing on DDIs, this, to a large extent will reduce possible complications and consequences (20, 22, 23).

## Experimental

This study was performed using Pardazesh Nosakh, a prescription processing software program, provided by the National Committee of Rational Drug Use. This program was developed for the DOS operating system and Novell Network in 1998. After a pilot run in the Medical University of Mashhad, the application was published for all Iranian Medical universities.

In this cross-sectional study, all data from March 21, 2007 to December 21, 2009 were analyzed. Data of the physicians› prescriptions were collected from 33 different medical science universities nationwide. Available data on prescriptions included physician identification, name, strength, and quantity of the medications dispensed. Due to the greater clinical importance of major DDIs, moderate and minor DDIs were not considered in this study.

From March 21, 2007 to March 20, 2008, 28, 956, 638 prescriptions were gathered. The number of prescriptions from March 21, 2008 to December 20, 2009 (in the spring, summer, and autumn) was 15, 610, 912. After processing and analyzing all of the prescriptions, the total occurrence of DDIs made by all physicians was determined and separated according to general practitioners and medical specialists. Data were from 14 different medical specialists consisting of internists, cardiologists, neurologists, psychiatrists, neurosurgeons, general surgeons, infectious diseases, urologists, dermatologists, ENT, ophthalmologists, orthopedists, and pediatrician. The results were then analyzed by methods that were applied in the book Drug Interaction Facts (DIF) (24). DIF rated DDIs in a five-item summary measure based on the severity and corresponding documentation (probable, suspected, possible, and unlikely) for each drug interaction.

## Results and Discussion

This study analyzed 28,956,638 prescriptions from March 21, 2007 to March 20, 2008 and 15, 610, 912 prescriptions from March 21, 2008 to December 20, 2009 from 33 different medical universities in Iran (Tables 1 and 3). 

**Table 1:** Result of processing and analyzing the total prescriptions

**2008-2009**					**2007-2008**					
**NP**	**%SI**	**NP**	**%MI**	**Total NP**	**NP**	**%SI**	**NP**	**%MI**	**Total NP**	
110,837	0.71	140,498	0.90	15,610,912	194,009	0.67	222,966	0.77	28,956,638	**All of physicians**
11,990	9.01	1 2,681	9.53	133,047	25,526	7.30	27,044	7.74	349,496	**Cardiologist**
60	0.04	80	0.06	137,947	164	0.06	217	0.07	297,314	**Dermatologist**
391	0.17	538	0.24	228,455	663	0.12	853	0.15	570,150	**ENT **
77,905	0.69	97,480	0.86	11,282,948	116,067	0.50	149,599	0.74	20,292,902	**General practitioners**
639	0.32	833	0.42	198,365	1,819	0.36	2,294	0.46	499,681	**General surgeon**
1,291	0.15	1,398	0.17	845,843	95	0.07	1180	0.08	1,450,666	**Gynecologist**
948	0.73	1,111	0.86	129,257	1,901	0.74	2149	0.83	257,951	**Infectious disease**
38,917	1.16	53,481	1.73	4,327,964	66,766	0.96	85,273	1.30	8,663,736	**All of medical specialists**
16,795	2.16	19,761	2.54	777,710	24,295	1.82	28,634	2.14	1,338,054	**Internist**
2,928	1.43	6,203	3.04	204,226	3,060	0.86	4,706	1.29	365,579	**Neurologist**
347	0.33	614	0.59	104,429	388	0.19	804	0.39	206,481	**Neurosurgeon**
129	0.05	165	0.07	137,947	395	0.07	460	0.09	535,621	**Ophthalmologist**
328	0.12	492	0.18	270,933	570	0.11	768	0.15	500,391	**Orthopedist**
815	0.11	1,011	0.13	774,807	1,862	0.11	2,158	0.13	1,695,170	**Pediatrist**
1,866	1.33	4,159	2.97	139,970	4,068	1.20	8,571	2.53	338,411	**Psychiatrist**
390	0.30	4,435	3.42	129,755	1,005	0.39	5,435	2.10	258,771	**Urologist**

NP: Number of Prescription; SI: Significant Interactions; MI: Major Interactions

A total of 20, 292, 902 prescriptions in 2007- 2008 and 11, 282, 948 prescriptions in 2008-2009 belonged to general practitioners and 8, 663,736 prescriptions in 2007-2008 and 4, 327, 964 prescriptions in 2008-2009 belonged to medical specialists. The percentage of interactions with significant clinical importance was higher in prescriptions of medical specialists and of those, cardiologists and internists ranked top on the list, while dermatologists ranked the lowest. The mean items per prescriptions (MIP) and percentage of prescriptions with more than four items per prescriptions (% > 4IP) are shown in Table 2. MIP and % > 4IP were highest in the cardiologists, general practitioners, internists and infectious disease specialists in 2007-2008, while MIP and % > 4IP were highest among cardiologists, infectious disease specialists, internists, and neurologists in 2008-2009.

**Table 2:** The mean items per prescriptions (M.I.P) and the percentage of prescriptions with more than four items per prescriptions (> 4IP) from -March 21, 2007 to -December 20, 2009

**2008-2009**		**2007-2008**			
**% > 4IP**	**M.I.P**	**% > 4IP**	**M.I.P**		
21	3.26	19		3.26	**All of physicians**
37	3.67	31		3.67	**Cardiologist**
3	2.06	4		2.13	**Dermatologist**
13	2.89	10		2.96	**ENT **
24	3.41	21		3.41	**General practitioners**
12	2.76	11		2.85	**General surgeon**
7	2.50	5		2.43	**Gynecologist**
25	3.42	15		3.12	**Infectious disease**
15	2.91	11		2.83	**All of medical specialists**
26	3.41	19		3.23	**Internist**
25	3.35	13		2.93	**Neurologist**
8	2.71	8		2.87	**Neurosurgeon**
13	2.89	2		2.22	**Ophthalmologist**
11	2.81	7		2.67	**Orthopedist**
15	2.98	11		2.94	**Pediatrist**
15	2.88	14		3.03	**Psychiatrist**
8	2.35	7		2.50	**Urologist**

The percentage of prescriptions with rapid onset interactions was highest among cardiologists. In contrast, the percentage of prescriptions with delayed onset interactions was the highest with psychiatrists in 2007-2008 and the highest in the neurologists in 2008-2009 (Table 3). The list of medications in Table 4 indicated that digoxin and diuretics were commonly associated with DDIs. The most common interacting combination prescribed was digoxin and furosmide in 2007-2008, and captopril and triamteren in 2008-2009 (Tables 5 and 7).

**Table 3:** Evaluation of prescriptions (Rapid and Delayed Onset in DDI_S_).

**2008-2009**				**2007-2008**				
**no** ^*^	**%Delayed**	**no** ^*^	**%Rapid**	**no** ^*^	**%Delayed**	**no** ^*^	**%Rapid**	**All of physicians**
1,405,481	8.8	498,120	2.54	2,385,690	7.51	734,601	2.16	
45,629	34.3	14,417	10.84	101,331	28.99	32,822	9.39	**Cardiologist**
1,382	1	105	0.08	2,870	0.97	421	0.14	**Dermatologist**
10,181	4.46	1,163	0.51	19,876	3.49	2,435	0.43	**ENT **
990,502	8.78	317,847	2.82	1,527,800	7.53	499,955	2.46	**General practitioners**
13,570	6.84	6,300	3.18	30,558	6.12	9,395	1.88	**General surgeon**
18,798	2.22	9,232	1.09	28,610	1.97	15,731	1.08	**Gynecologist**
16,007	12.38	5,233	4.05	24,622	9.55	6,184	2.4	**Infectious disease**
497,652	16.09	108,637	2.74	785,614	12.57	151,502	2.06	**All of medical specialists**
127,679	16.42	48,744	6.27	181,861	13.59	47,471	3.55	**Internist**
119,345	58.44	11,128	5.45	117,739	32.21	9,017	2.47	**Neurologist**
16,866	16.15	988	0.95	33,214	16.09	2,378	1.15	**Neurosurgeon**
8,600	3.4	497	0.2	15,165	2.83	1,183	0.22	**Ophthalmologist**
16,766	6.19	872	0.32	14,284	2.85	1,547	0.31	**Orthopedist**
18,359	2.37	3,037	0.39	36,165	2.13	6,733	0.4	**Pediatrist**
70,524	50.39	4,288	3.06	155,780	46.03	9,824	2.9	**Psychiatrist**
13,946	10.75	2,633	2.03	23,539	9.10	6,361	2.46	**Urologist**

no^*^: number of prescriptions

**Table 4:** Short list of common interacting medications

*Digoxin*
Diuretics
HMG CoA reductase Inhibitors
Allopurinol
ACE Is
Warfarin
Gemfibrozil
Haloperidol
Amiodarone
Clonidine

**Table 5:** Top of 10 DDIs pairs by incidence per 100 prescriptions from March 21, 2007 to march 20, 2009.

**Drug 2**	**Drug 1**	**%**	**no** ^*^
Digoxin	Furosmide	0.11	74,084
Captopril	Triamterene-H	0.09	67,619
Gemfibrozil	Atrovastatin	0.08	65,103
Haloperidol	Propranolol	0.04	26,789
Amitriptyline	Clonidine	0.03	20,666
Doxycycline	Penicilline G	0.01	8,526
Chlorpromazine	Propranolol	0.01	8,049
Propranolol	Verapamil	0.009	6,904
Amiodaron	Digoxin	0.008	6,417
Azithromycine	Atrovastatin	0.007	5,727

no^*^: number of prescriptions

**Table 6:** Abundance of drug categories in the drug interactions

**%Incidence per total of prescriptions**	**drug categories**
85	Cardiovascular
74	Anti hypertensive
24.78	ACEI s
34.44	Antiarrhythmic
22.63	Digitalis
30.08	CNS drugs
4	Diuretics
1	Macrolides
1	Tetracyclins
1	Penicillins

**Table 7:** The results of investigation of major DDIS from march 21, 2007 to December 20, 2009

**Documentation**	**Total of prescriptions**	**no**_ 2_^*^	**no **_1_^*^	**Onset**	**Significance**	**Severity**	**Drug 2**	**Drug 1**
probable	74,084	29,901	44,183	Delayed	1	Major	Furosemide	Digoxin
probable	64,063	24,406	39,657	Delayed	1	Major	Triamterene	Captopril
suspected	65,083	29,543	35,540	Delayed	1	Major	Atorvastatin	Gemfibrozil
probable	59,640	27,962	31,678	Delayed	1	Major	Triamterene	Enalapril
possible	39,497	14,650	24,847	Rapid	4	Major	Codeine	Cimetidine
suspected	38,075	14,369	23,706	Delayed	1	Major	Lovastatin	Gemfibrozil
possible	26,789	11,679	15,110	Rapid	4	Major	Propranolol	Haloperidol
unlikely	25,205	11,198	14,007	Delayed	5	Major	Hydrochlorothiazide	Allopurinol
probable	21,706	7,919	13,787	Delayed	1	Major	Spironolactone	Captopril
probable	20,666	9,040	11,626	Rapid	1	Major	Clonidine	Amitriptyline
probable	11,586	4,703	6,883	Delayed	1	Major	Hydrochlorothiazide	Digoxin
possible	9,756	3,729	6,027	Rapid	4	Major	Fluoxetine	Metoclopramide
suspected	8,526	2,906	5,620	Delayed	1	Major	Penicillin G	Doxycycline
probable	8,326	3,237	5,089	Delayed	1	Major	Spironolactone	Enalapril
probable	8,049	3,008	5,041	Delayed	1	Major	Propranolol	Chlorpromazine
suspected	7,365	2,621	4,744	Delayed	1	Major	Simvastatin	Gemfibrozil
probable	6,904	2,509	4,395	Rapid	1	Major	Verapamil	Propranolol
probable	6,417	2,372	4,045	Delayed	1	Major	Digoxin	Amiodarone
probable	7,818	4,004	3,814	Delayed	1	Major	Atorvastatin	Azithromycin
suspected	5,991	2,396	3,595	Delayed	1	Major	Nortriptyline	Cisapride
suspected	6,717	3,488	3,229	Delayed	1	Major	Sulfasalazine	Methotrexate
possible	4,651	1,548	3,103	Rapid	4	Major	Trazodone	Fluoxetine
suspected	4,596	1,656	2,940	Delayed	2	Major	Warfarin	Penicillin G
possible	4,219	1,427	2,792	Delayed	4	Major	Captopril	Allopurinol
probable	4,620	1,913	2,707	Delayed	1	Major	Lovastatin	Azithromycin
probable	4,107	1,448	2,659	Rapid	1	Major	Verapamil	Atenolol
possible	5,395	2,764	2,631	Rapid	4	Major	Omeprazole	Methotrexate
suspected	7,266	4,929	2,237	Delayed	1	Major	Naproxen	Methotrexate
suspected	3,352	1,239	2,113	Delayed	1	Major	Trifluoperazine	Cisapride
suspected	3,510	1,410	2,100	Delayed	1	Major	Propranolol	Clonidine
established	3,471	1,450	2,021	Delayed	1	Major	Warfarin	Aspirin

no_1_*: Number of prescriptions with the major DDIs from March 21, 2007 to march 20, 2008; no_2_*: Number of prescriptions with the major DDIs from March 21, 2007 to December 20, 2009

Our findings also revealed the most alarming DDIs in prescriptions from March 21, 2007 to December 20, 2009 with significant clinical importance by incidence per 100 prescriptions as shown in Table 5.

The study of DDIs in the present population showed that the highest DDIs with clinical importance were the antihypertensive drugs such as beta blockers, calcium channel blockers followed by ACE-Is (Table 6).

Based on these results, the increase in DDIs in the prescriptions occurs when number of items per prescription increased, which led to a rise in the index of DDIs with clinical significance.

**Figure 1:**
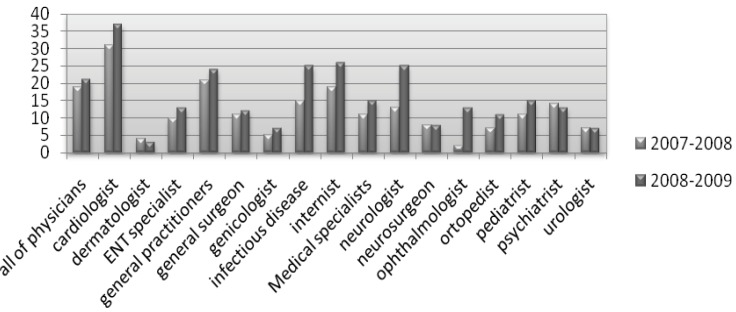
The percentage of prescriptions with more than four items per prescription in the years 2008-2009 as compared to 2007-2008

**Figure 2:**
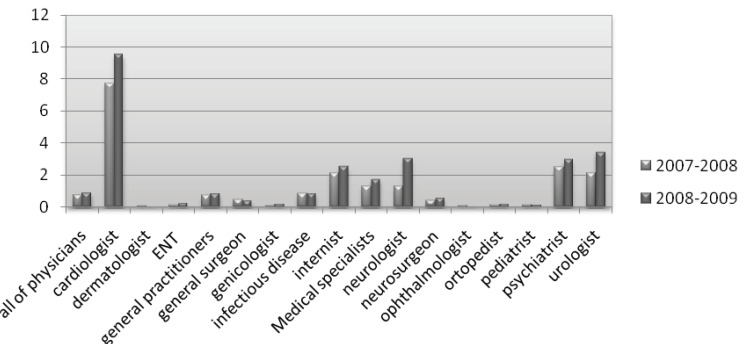
Major drug interactions in the prescriptions of all physicians in the years 2008-2009 as compared to 2007-2008

This study revealed that with the increase of percentage in prescriptions with more than four items per prescription in the years 2008-2009, as compared to 2007-2008 (Figure 1), significant major DDIs in the prescriptions of all physicians, general practitioners, and medical specialists in 14 different fields significantly increased. (Figure 2) This was clearly visible in the prescriptions of cardiologists, internists, General practitioners, infectious disease specialists and neurologists who had a greater number of items per prescription.

Polypharmacy is an important factor which leads to DDIs; however, the more the number of items per prescriptions, the more the likelihood of DDI’s occurance. Straubhaar *et al. *(25) reported in a study at the University Hospital Basel that hospitalization of patients with heart failure resulted in an increase in the number of drugs prescribed per patient and, thereby, also in the number of potentially interacting drug combinations per patient. During the hospital stay, close medical monitoring combined with continuous nursing and therapeutic care is generally guaranteed, but this may profoundly change after discharge. The elderly generally have several concurrent diseases (26) and consequently, the number of drugs used to treat them is greater, and the greater the number of drugs, the higher the possibility of DDIs. Malone *et al. *(27), using the prescription database of a North American insurance company with 46 million clients, carried out a retrospective study of the prevalence of 25 clinically relevant interactions. They found that their clients of 70 years or more made up to 7.1% of the total population, but they suffered from 44.8% of all DDIs.

In three past studies on primary care, the rates of potential DDIs for patients receiving two or more drugs were 24.3%, 29.5%, and 42.0%, respectively (28, 39). Another study reported 2.2% of prescriptions with adverse interacting drugs in relation to all prescriptions (30, 31).

In another study, all drug pairs concurrently prescribed to 9481 adults aged 50 to 75 years were evaluated in a health-screening examination. More than 52% of the patients received a combination therapy of drug pairs and 881 (6.4%) were identified as interacting. Of these 881 interactions, 132 (15.0%) were of major severity and 101 of 132 (76.5%) were considered manageable. Only 31 (23.5%) of 132 major interactions (3.5% of all interacting pairs) offered no management options and should thus have been avoided (32). The results of this study and past studies, especially on DDIs in Iran when compared to the present, showed that its percentage is rising and continues to pose problems (33-36).

In primary health care, 9-70% of patients are reported to be exposed to drugs with the risk of a drug interaction, with 1-23% of major significance (37-42). A French study reports an incidence of 27 per 10,000 prescriptions with contraindicated DDIs in an ambulatory outpatient population (43). During hospital admission, the number of DDIs per patient increased with potential clinically relevant DDIs occurring in 1 out of 70 prescriptions (44-47). In another study, 22 potential DDIs of clinical relevance and 65 of ‘possible’ clinical relevance per 100 outpatients per year were recorded. Reported incidences in outpatients ranged from 9.2% to 70.3% for DDIs of any severity and from 1.2% to 23.3% for those considered of major significance (31, 1, 48-51).

In addition, Chen *et al. *(52) found an incidence of 1.9 per 1000 patient (95% confidence interval 1.5, 2.3) of prescribed potentially hazardous/contraindicated DDIs. They identified multiple possible causes (*e.g.*, lack of knowledge of the DDIs or of the patient medication history) and system failures (*e.g.*, incomplete medication records, lack of communication between primary and secondary care or between the prescriber and the patient) for the dispensing of contraindicated drug combinations.

Unfortunately, there are little supportive data on the incidence of potential DDIs in other large ambulatory populations. Few published studies have determined such errors exclusively, with most aggregating various types of potential medication errors.

**Figure 3:**
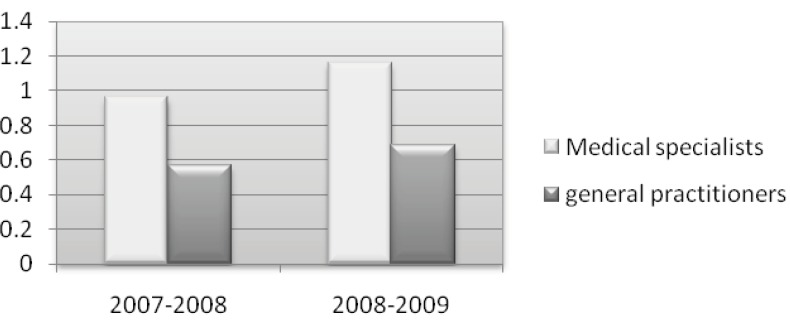
Major drug interactions with significant clinical importance in the prescriptions of general and specialist practitioners

**Figure 4:**
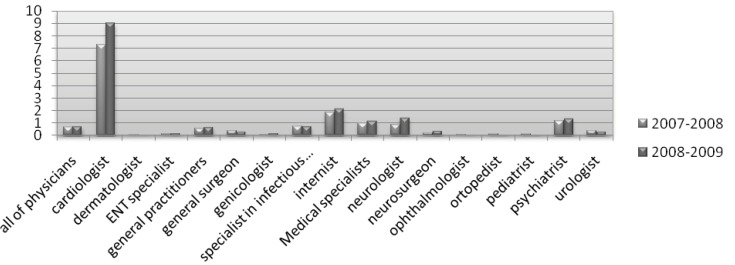
Major drug interactions with significant clinical importance in the prescriptions of all physicians in the years 2008-2009 as compared to 2007-2008

Quite frequently, the determination of medication errors found in studies has been part of a planned intervention that might include medication selection or dosage assessment (53-55), laboratory monitoring (54-56), or inappropriate prescribing (56, 59). Moreover, most studies on medication errors have involved interventions in hospitalized or with institutionalized patients, but not with outpatients (54, 56, 60-64). In the year 1988, Dumbro *et al*. (28) reported that out of the total number of DDIs they had discovered, 17% were of great significance, while in our study, we found major DDIs with significant clinical importance as shown in Table 2. Comparing general practitioners and medical specialists, it is evident that from March 21, 2007 to December 20, 2009, more alarming DDIs were found in the prescriptions of medical specialists (Figure 3). The same observation was made when we compared the prescriptions resulting in major DDIs of clinical importance (Figure 4). It seems that medical specialists deal more with potent drugs with a low therapeutic index that might be a cause of more DDIs in their prescriptions.

**Figure 5:**
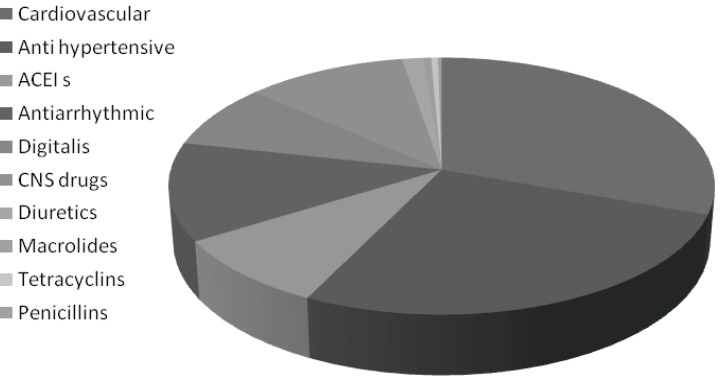
Abundance of drug categories in the DDIs

The results of our study also revealed that the most common drugs associated with major interactions of significant clinical importance were those prescribed by cardiologists. Previous studies have also shown that prescriptions of cardiologists had the highest rate of DDIs (65). With regard to the interactions detected, other studies without intervention by pharmacists found more DDIs in the fields of cardiology (27.9%), hematology (23.4%), neurology (2.7%), psychiatry (5.3%), and gastroenterology (5.1%). In addition, 151 DDIs were detected during the three-month follow-up period. The interactions found most frequently were: digoxin-furosemide, furosemid-corticoid, AAS-low molecular weight heparin, amiodarone-furosemide, omeprazole-diazepam, phenytoin-corticoid and AAS-oral antidiabetics (65, 66), while in our study, we found that the most common interacting combination prescribed were: digoxin-furosmide, captopril-plustriamteren, gemfibrozil-atorvastatin, enalapril-triamteren, cimetidine-codeine, gemfibrozil-lovastatin, and haloperidol-propranolol.

Shivo *et al*. (67) in the year 2000 carried out a study in Finland that showed harmful interactions between over the counter (OTC) drugs (especially NSAIDs and analgesics) and prescription drugs. This shows that DDIs do not solely occur with prescription drugs, but food and other OTC drugs purchased and consumed by patients also play a role and could sometimes be responsible for treatment failure.

## Conclusions

Potential drug interactions are frequent among outpatients prescribed multiple medications and the rate is directly related to the number of drugs prescribed. It is our opinion that physicians must pay closer attention to DDIs, especially cardiovascular, antihypertensive, ACEIs, antiarrhythmic, digitalis, and CNS drugs and diuretics and the DDIs between OTC drugs and prescription drugs, especially in terms of side effects and the economic burden that they may produce. Moreover, adhering to the correct policies of writing prescriptions, being up-to-date on drug information, and participating in related educational classes and workshops by physicians, may significantly increase the chances of the appropriate drug being selected for treatment and hence quicker patient recovery .
